# Mutations of *PDS5* genes enhance TAD-like domain formation in *Arabidopsis thaliana*

**DOI:** 10.1038/s41467-024-53760-x

**Published:** 2024-10-29

**Authors:** Anna-Maria Göbel, Sida Zhou, Zhidan Wang, Sofia Tzourtzou, Axel Himmelbach, Shiwei Zheng, Mónica Pradillo, Chang Liu, Hua Jiang

**Affiliations:** 1 Department of Epigenetics, Institute of Biology, University of Hohenheim, Garbenstrasse 30, Stuttgart, Germany; 2 Institute for Biochemistry and Biology, University of Potsdam, Karl-Liebknecht-Str. 24-25, Potsdam-Golm, Germany; 3 Leibniz Institute of Plant Genetics and Crop Plant Research (IPK), Gatersleben, Germany; 4 Max Planck Institute of Molecular Plant Physiology, Potsdam-Golm, Germany; 5 Departamento de Genética, Fisiología y Microbiología, Facultad de Ciencias Biológicas, Universidad Complutense, Madrid, Spain

**Keywords:** Chromosomes, Epigenomics, Plant genetics, Chromosomes

## Abstract

In eukaryotes, topologically associating domains (TADs) organize the genome into functional compartments. While TAD-like structures are common in mammals and many plants, they are challenging to detect in *Arabidopsis thaliana*. Here, we demonstrate that *Arabidopsis* PDS5 proteins play a negative role in TAD-like domain formation. Through Hi-C analysis, we show that mutations in *PDS5* genes lead to the widespread emergence of enhanced TAD-like domains throughout the *Arabidopsis* genome, excluding pericentromeric regions. These domains exhibit increased chromatin insulation and enhanced chromatin interactions, without significant changes in gene expression or histone modifications. Our results suggest that PDS5 proteins are key regulators of genome architecture, influencing 3D chromatin organization independently of transcriptional activity. This study provides insights into the unique chromatin structure of *Arabidopsis* and the broader mechanisms governing plant genome folding.

## Abstract (web summary)

Topologically associating domain (TAD) facilitates the creation of distinct chromatin compartments and specific chromatin interaction, but TAD-like structures are lacking in *Arabidopsis*. Here, the authors report the possible role of *PDS5s* in suppressing the formation of TAD-like domains in *Arabidopsis*.

## Introduction

The three-dimensional organization of chromatin is essential for gene expression, DNA replication, and damage repair. In mammals, Hi-C studies have revealed hierarchical genome structures, notably identifying topologically associating domains (TADs) as key features^1^. TADs are characterized by strong internal chromatin interactions and reduced contacts with neighboring regions^1^. Genes residing within the confines of a single TAD often exhibit similar expression patterns, enabling coordinated regulatory control. TAD boundaries, marked by insulators, prevent unnecessary interactions between elements like enhancers in adjacent TADs, ensuring precise gene regulation. The spatial proximity within TADs enhances interactions among genes and regulatory elements, affecting chromatin accessibility and gene transcription^2^. Disruptions in TAD distribution, often due to genomic rearrangements, can lead to misregulated genes and are linked to diseases such as cancer and developmental disorders^3–5^. There are two mechanisms for establishing TADs. The first type, which shows particularly strong chromatin contacts between TAD borders, involves cohesin and CTCF protein complexes. They create looped structures by extruding chromatin loops until they reach a pair of oppositely oriented CTCF binding sites overlapping with TAD boundaries^6–9^. The second type of TAD, where the boundary and intra-TAD contact strengths are similar, is shaped by gene expression, especially at TAD borders. This type of TAD also correlates with the epigenomic landscape^10,11^.

Surprisingly, TADs, which typically range in size from dozens to hundreds of kilobase pairs, are not prominent in the *Arabidopsis* genome^12–14^. Nonetheless, with deep sequencing of *Arabidopsis* Hi-C libraries, or alternative approaches that resolve chromatin interactions at high resolution (e.g., sub-kb), it has been revealed that the *Arabidopsis* genome forms chromatin domains, whose borders correlate tightly with chromatin accessibility, epigenetic marks, and gene boundaries^15–18^. On the contrary, conspicuous TAD structures (also referred to as “TAD-like domains” in the plant science community) were found in many model species across the plant kingdom (reviewed by Domb et al.^19^). The absence of TAD-like domains in *Arabidopsis* has puzzled researchers, with theories suggesting it may be due to the lack of insulator proteins, small genome size, short intergenic regions, and high gene density^19–21^.

In this work, we show that *Arabidopsis* PDS5 proteins play a negative role in the formation of TAD-like domains. Through Hi-C analysis, we demonstrate that mutations in *PDS5* genes lead to the widespread emergence of enhanced TAD-like domains throughout the *Arabidopsis* genome, excluding pericentromeric regions. These domains exhibit increased chromatin insulation and enhanced chromatin interactions, yet no significant changes in gene expression or histone modifications. Our findings suggest that PDS5 proteins are key regulators of genome architecture, influencing 3D chromatin organization independently of transcriptional activity, providing further insights into the unique chromatin structure of *Arabidopsis*.

## Results

### Enhanced TAD-like domains in *Arabidopsispds5* mutants

Cohesin complexes in plants and animals have conserved function in regulating chromosome segregation during cell division^22^. In mammals, cohesin is also indispensable to define 3D genome organization. We conducted Hi-C experiments to assess the potential impact of the perturbation in cohesin activities on the *Arabidopsis* 3D genome (Supplementary Data 1). Since loss-of-function mutants of *Arabidopsis* cohesin subunits SMC1 and SMC3 are not viable, we selected mutants that potentially affect cohesin-chromatin interactions^23^. To this end, *wapl1 wapl2* double mutants (hereafter referred to as *wapl1/2*) and *pds5a pds5b pds5c pds5e* quadruple mutants (hereafter referred to as *pds5a/b/c/e*) were used for Hi-C experiments^8,24,25^.

**Fig. 1:**
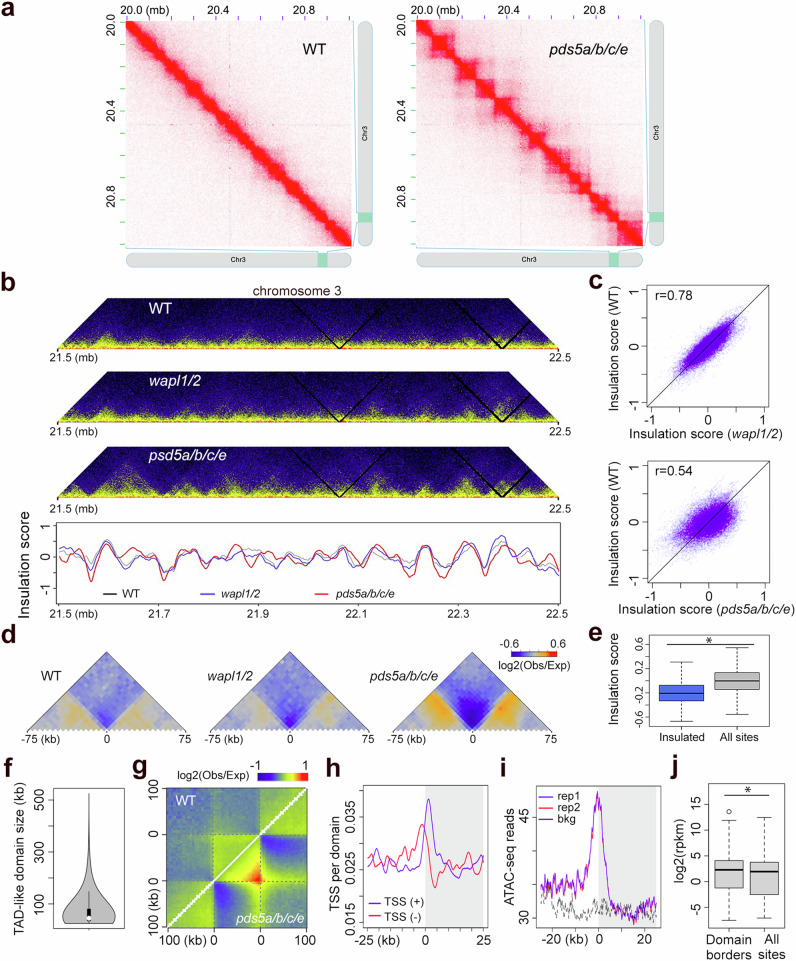
Analysis of TAD-like domains in *pds5a/b/c/e.* **a** Prominent TAD-like structures in *pds5a/b/c/e*, showcased by a representative 1 Mb genomic region. **b** Comparison of insulation score profile of a 1 Mb region at chromosome 3 with the corresponding Hi-C map shown on top. The regions showing local insulation score minima indicate strong chromatin insulation. **c** Genome-wide correlation of insulation scores between different plants. r, Pearson correlation coefficients. **d** Metagene plots of chromatin interaction patterns in different plants around the loci displaying strong chromatin insulation in *pds5a/b/c/e*. **e** Insulation scores in wild-type plants. The category “Insulated” refers to those loci showing strong chromatin insulation in *pds5a/b/c/e*. *, significant (*p* = 4.2 × 10^−114^) according to a two-sided Mann–Whitney U-test. The boxplots depict the data quartiles (first and third), the dashed lines represent individual data points, and the median is indicated by black lines within the boxes. **f** Size distribution of TAD-like domains in *pds5a/b/c/e*. The boxplot follows the same format as in (**e**). **g** Metagene plot illustrates relative chromatin contact strengths in TAD-like domains identified in *pds5a/b/c/e*. The flanking regions (100 kb) are also included. The top side shows WT and the bottom side shows mutant. Besides the strong insulation region, the extended signal on the top of the triangle promotes the detection of strong boundaries in *pds5a/b/c/e*. **h** Distribution of TSS around *pds5a/b/c/e* TAD borders (labeled with 0). The right gray side represents the 25 kb within the TAD-like domains, the left side from 0 represents 25 kb of flanking area. Both transcription directions are included. **i** Chromatin accessibility associated with TAD-like domain borders in *pds5a/b/c/e*. **j** Comparison of gene expression in *pds5a/b/c/e* between genes located at TAD-like domain borders and elsewhere. *, significant (*p* = 6.2 × 10^−13^) according to a two-sided Mann–Whitney U-test. The boxplots follow the same format as in (**e**). Source data are provided as a Source Data file.

At the chromosomal level, the Hi-C maps of *wapl1/2*, *pds5a/b/c/e*, and wild-type (WT) appeared similar (Supplementary Fig. 1a and Supplementary Fig. 2). However, closer inspection of the diagonal revealed striking differences in local chromatin contacts (i.e., contacts that span distances up to a few hundred kilobase pairs) in the *pds5a/b/c/e* mutants (Fig. 1a, Supplementary Figs. 1a–c). In *pds5a/b/c/e*, TAD-like domains were clearly visible and widely distributed through the genome, except in the pericentromeric regions, which remained constitutive heterochromatin. Consistently, chromatin interaction decay plots showing how contact strength decreases with genomic distance revealed specific changes in chromosome arm regions of *pds5a/b/c/e* compared to WT, indicating that the former adopted distinct chromatin packing patterns (Supplementary Fig. 1d).

The *Arabidopsis* genome encodes five homologs of *PDS5*, which are redundantly involved in not only chromatid cohesion and DNA damage repair but also small interfering RNA production and transcriptional regulation^26–29^. At the transcriptional level, *PDS5A* was the most actively expressed *PDS5* gene, with its expression level approximately six times higher than that of its closest homolog, *PDS5B* (Supplementary Data 2, 3). Compared to other PDS5 proteins, PDS5A and PDS5B contain substantially more Armadillo-type fold domain regions, which potentially enhance their interactions with proteins and nucleic acids^27^. To investigate the potential functional redundancy of *Arabidopsis PDS5* genes in regulating chromatin organization, we conducted further Hi-C experiments to examine single, double, and triple *pds5* mutants. All examined mutants containing the *pds5a* allele exhibited similar TAD-like domains as those in *pds5a/b/c/e*, indicating that *PDS5A* is the major factor suppressing TAD-like structures (Supplementary Fig. 3a). Supporting this notion, transforming *pds5a/b* with a construct bearing *PDS5A* genomic DNA restored its chromatin organization patterns (Supplementary Figs. 3b–d). Therefore, we conclude that PDS5A is the main regulator among all *PDS5* genes in *Arabidopsis*.

### *pds5* TAD-like domains do not alter other chromatin features

Given that *PDS5s* redundantly regulate *Arabidopsis* seedling development^27^ and that *pds5a/b/c/e* exhibited the most altered transcriptome profile (Supplementary Fig. 4 and Supplementary Data 4–7), we presumed that chromatin organization was most affected in *pds5a/b/c/e* compared to other lower-order mutants. Thus, we deep-sequenced the Hi-C libraries from *pds5a/b/c/e* for further analyzes (Supplementary Data 1). When compared to WT, approximately 15% of the chromatin in *pds5a/b/c/e* exhibited AB compartment swaps, indicating mild rewiring of genome topology at the chromosomal level (Supplementary Figs. 5a–d and Supplementary Data 8). Interestingly, these AB compartment swaps were not associated with changes in active (e.g., H3K4me3) or repressive (e.g., H3K27me3 and H3K9me2) histone marks, which were enriched in A and B compartment regions, respectively (Supplementary Fig. 5b–f and Supplementary Data 9-14). Furthermore, while A and B compartment identities are typically linked to active and inactive gene expression, the switch in compartment identity in *pds5a/b/c/e* occurred independently of transcriptomic changes (Supplementary Figs. 5g, h).

Next, we examined the newly emerged local chromatin contact patterns on the Hi-C map of *pds5a/b/c/e*. By calculating insulation scores, we identified genomic regions within *pds5a/b/c/e* that exhibited strong chromatin insulation (Fig. 1b and Supplementary Data 15). Notably, most of the insulated regions in *pds5a/b/c/e* already displayed weak insulation patterns in WT plants (Fig. 1c–e). Consistent with a recent report, these insulated regions were enriched with 5’ flanking regions of gene transcription start sites^16^ (Supplementary Fig. 6). We also annotated TAD-like domains in *pds5a/b/c/e* (Fig. 1f and Supplementary Data 16), which showed a weak tendency of domain formation in the WT Hi-C map (Fig. 1g). Similar to other plant species, *the* borders of TAD-like domains in *pds5a/b/c/e* were enriched with promoter regions, higher chromatin accessibility, and genes exhibiting higher expression levels (Fig. 1h–j and Supplementary Fig. 7a). This suggests that the emergent TAD-like domain structures are associated with gene expression^30^. However, further examination of transcriptional activities in these regions, including euchromatin mark deposition (H3K4me3), chromatin accessibility, RNA-polymerase II association, and gene expression levels, did not reveal noticeable changes in *pds5a/b/c/e* (Supplementary Fig. 8). Therefore, the changes in chromatin insulation observed in *pds5a/b/c/e* did not have a direct impact on gene expression.

### Reorganized long-range chromatin loop network in *pds5*

**Fig. 2:**
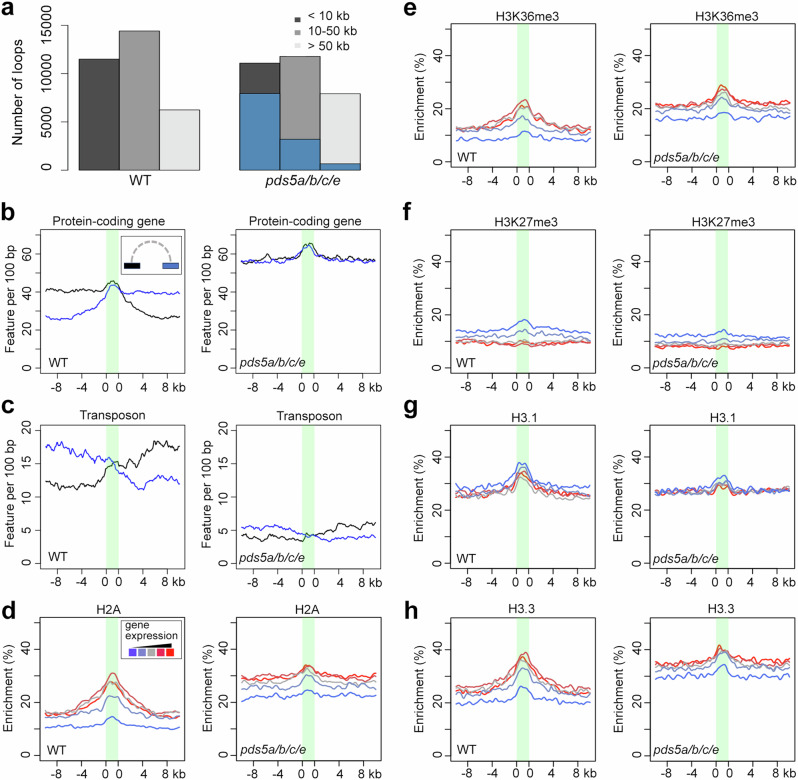
Comparison of epigenetic features associated with chromatin loops and gene expression between WT and *pds5a/b/c/e.* **a** Proportion of chromatin loop sizes in WT and *pds5a/b/c/e*. Loops smaller than 10 kb, between 10 and 50 kb, and bigger than 50 kb are included. Blue scaled bars indicate those loops in *pds5a/b/c/e* which are also identified in wild-type plants. **b**, **c** Genomic features at regions annotated with chromatin loops, which are illustrated as the green area in each plot. The blue and black curves refer to individual loop anchor regions. **d**–**h** Enrichment of epigenetic marks around chromatin regions that form loops with protein-coding genes. These regions are divided into five groups according to expression levels of their interacting protein-coding genes. The color gradient from blue via gray to red represents ascending gene expression.

Along with the emergence of TAD-like domains, extensive changes in local chromatin contacts occurred in *pds5a/b/c/e*. To better understand the chromatin interaction network in *pds5a/b/c/e*, we identified chromatin contacts with statistical significance (referred to as “chromatin loops”) in WT and mutant plants^31^. Both genotypes exhibited similar chromatin loops at short distances (<10 kb), but the fraction of shared chromatin loops decreased dramatically at longer distances (10–100 kb) (Fig. 2a). Among the long-distance chromatin loops (10–100 kb), approximately 40% of loop-forming regions in WT plants overlapped with protein-coding genes, whereas this percentage rose to 65% in *pds5a/b/c/e* mutants, partly due to a lower occurrence of transposons overlapping with *pds5a/b/c/e* loops (Fig. 2a, b).

For the long-distance chromatin loops in *pds5a/b/c/e*, we examined their epigenetic profiles in relation to gene expression. Currently, comprehensive datasets that depict various epigenetic marks in *pds5a/b/c/e* are lacking. However, since WT and *pds5a/b/c/e* plants exhibited highly similar patterns of three representative epigenetic marks (i.e., H3K4me3, H3K9me2, and H3K27me3), and since both groups had comparable transcriptome profiles (Supplementary Figs. 7b and 9), we expected the epigenomic landscape of *pds5a/b/c/e* to be similar to that of WT plants. Based on this assumption, we analyzed several histone marks at loci forming chromatin loops with protein-coding genes, using the epigenetic features of WT seedlings to approximate those in *pds5a/b/c/e*. We found that in both WT and *pds5a/b/c/e* plants, inactive genes tended to form chromatin loops with regions carrying repressive chromatin marks (e.g., H3K27me3), while actively expressed genes preferentially interacted with active chromatin regions (e.g., those decorated with H3K36me3 and H2A) (Fig. 2d–h). These results suggest that *pds5a/b/c/e* and WT chromatin have reconstituted long-range chromatin contact networks according to the same chromatin segregation principle, whereby active and repressed chromatin form partners with similar epigenetic profiles.

## Discussion

Our findings reveal that PDS5A, likely working in conjunction with other PDS5 homologs, plays a key role in modulating long-range chromatin contacts in *Arabidopsis*. The presence of PDS5A leads to the genome-wide suppression of TAD-like structures, making them hardly recognizable on Hi-C maps (Fig. 1a). Although we did not extensively profile individual epigenetic marks, the emergence of TAD-like domains in *pds5a/b/c/e* mutants appears independent of these marks, as histone modifications such as H3K4me3, H3K9me2, and H3K27me3 remained largely unchanged. This raises important questions about the mechanisms underlying TAD-like domain suppression in *Arabidopsis*, which future research will need to address. One possible explanation for the role of PDS5A in genome organization lies in its Tudor domain^29,32^, which exhibits high sequence and structural similarity to PDS5C, a protein known to recognize mono-methylated histone H3K4 (Supplementary Fig. 10). This suggests that PDS5A may interact with chromatin through this domain, potentially influencing genome topology. Identifying PDS5A as a major factor in *Arabidopsis* genome organization sheds light on other structural proteins that might also be involved in regulating plant 3D chromatin architecture.

Several studies demonstrated that TADs were critical for gene expression regulation, as disruption of TAD border resulted in misregulation of genes located nearby^5,33,34^. However, it seems that only a subset of TADs is crucial for specific enhancer-promoter interactions, because many large-scale genomic studies indicate that changes to TAD domains often result in only marginal alterations in gene expression^10,11,35–38^, challenging the idea that TAD structures are universally critical for gene regulation. A question about the interplay between gene expression and chromosome topology still remains to be resolved^39–41^. Our analysis of *Arabidopsis pds5* mutants demonstrates that TAD-like structures can form without significant alterations in epigenetic landscapes or gene expression (Supplementary Fig. 8). This observation suggests that the *Arabidopsis* 3D genome architecture and its local chromatin feature (e.g., individual gene expression) are largely uncoupled. Nonetheless, it should be noted that bulk transcriptomic analyzes, which we used in this study, may not fully capture the regulatory influence of TADs on gene expression. Methods resolving variation amongst individual cells, such as single-cell transcriptomic techniques, have shown that cohesin can influence the co-expression of cis-linked genes at the single-cell level^35^. Similar regulatory mechanisms could be present in *Arabidopsis pds5* mutants, but further research is needed to verify this.

In conclusion, the *Arabidopsis PDS5A* gene provides us a genetic basis for why this plant lacks TAD-like domains, unlike other plant species. We envisage that further research into PDS5 and related proteins in other plant species will deepen our understanding of plant genome organization and reveal how different plants may regulate their 3D genome architecture in diverse ways. This could also enhance our broader understanding of chromatin dynamics and its relationship with gene regulation across various plant species.

## Methods

### Plant materials and growth conditions

The *Arabidopsis thaliana* accession *Columbia* was used as wild-type (WT) reference. The *pds5a*, *pds5b*, *pds5c*, *pds5e*, *pds5a/b*, *pds5a/b/c, pds5a/b/c/e* and *wapl1/2* mutants were previously reported^27,42^. Seeds were treated at 4 °C for 3 days before sowing on the soil. All plants were cultured in the glasshouse with a 16 h: 8 h, light: dark photoperiod at 22 °C.

### Plasmid construction

To make the *PDS5A:2HA* construct, a 10 kb genomic DNA containing the RNA-coding region of *PDS5A* was amplified as two overlapping fragments: oligos 5’- TGTACAAAAAAGCAGGCTTTCGACGTCGGTATTCTTACACTAATG -3’ and 5’- AGGGTATCCAGCATAATCTGGTACGTCGTATGGGTATCCTATTGCTGTCCTCGAGATTGA -3 for the first fragment, while oligos 5’- CTTTGTACAAGAAAGCTGGGTCAAGAATAAGTAGTTGACATCA -3’ and 5’- GATTATGCTGGATACCCTTACGACGTACCAGATTACGCTTAGGTTTGCCGTATGCCGTA -3’ for the second fragment. The PCR products were cloned into the pFK206 vector^43^ via a Gibson Assembly reaction (NEB). The construct sequence was verified with Sanger sequencing and transferred into plants with the Agrobacteria-mediated floral dip method.

### In situ Hi-C and data analyzes

In situ Hi-C libraries were generated following established protocols^44,45^. Each sample was subjected to two Hi-C library replicates, with approximately 0.5 g of fixed sample homogenized for nuclei isolation. The isolated nuclei were resuspended in 150 µL of 0.5% SDS and divided into three tubes. After a 5-minute incubation at 62 °C for penetration, SDS was neutralized by adding 145 µL water and 25 µL 10% Triton X-100, followed by a 15-minute incubation at 37 °C. Subsequently, chromatin was digested using 50 U Dpn II (NEB) in each tube overnight at 37 °C. The following day, Dpn II was inactivated by a 20-minute incubation at 62 °C. Sticky ends were then filled in by adding 1 µL of 10 mM dTTP, 1 µL of 10 mM dATP, 1 µL of 10 mM dGTP, 10 µL of 1 mM biotin-14-dCTP, 29 µL water, and 40 U Klenow fragment (Thermo Fisher), with an incubation at 37 °C for 2 h. Following the addition of 663 µL water, 120 µL of 10x blunt-end ligation buffer (containing 300 mM Tris-HCl, 100 mM MgCl2, 100 mM DTT, 1 mM ATP, pH 7.8), and 40 U of T4 DNA ligase (Thermo Fisher), proximity ligation was conducted at room temperature for a duration of 4 h. Subsequently, the ligation products from the three tubes of identical samples were combined and resuspended in 650 µL SDS buffer (50 mM Tris-HCl, 1% SDS, 10 mM EDTA, pH 8.0). Following treatment with 10 µg proteinase K (Thermo Fisher) at 55 °C for 30 minutes, de-crosslinking was performed by adding 30 μL 5 M NaCl and incubating overnight at 65 °C. The DNA was recovered and treated with RNase A at 37 °C for 30 minutes. After purification, 3~5 μg of the recovered Hi-C DNA was adjusted to 130 μL with TE buffer (10 mM Tris-HCl, 1 mM EDTA, pH 8.0), and sheared using a Q800R3 (QSONICA) sonicator with the following settings: 25% amplitude, 15 s ON, 15 s OFF, pulse-on time for 4.5 min, to achieve a fragment size shorter than 500 bp. The sonicated DNA was purified with Ampure beads to recover fragments longer than 300 bp. Subsequently, in a 50 μL reaction volume, the DNA was mixed with 0.5 μL 10 mM dTTP, 0.5 μL 10 mM dATP, and 5 U T4 DNA polymerase, and incubated at 20 °C for 30 minutes to remove biotin from unligated DNA ends. Following purification with Ampure beads, the DNA underwent end-repair and adapter ligation using the NEBNext® Ultra™ II DNA Library Prep Kit (NEB). Following affinity purification using Dynabeads MyOne Streptavidin C1 beads (Invitrogen), the ligated DNA was subsequently subjected to amplification through 12 PCR cycles. The resulting libraries were then sequenced using an Illumina Novaseq instrument, generating reads with a length of 2 × 150 bp.

Reads mapping to the TAIR10 genome with Bowtie 2 (v2.2.4), removal of PCR duplicates, and reads filtering were performed as described. Hi-C reads of each sample are summarized in Supplementary Data 1. Hi-C map normalization was performed by using an iterative matrix correction function in the “HiTC” package in R programm. For all Hi-C maps, the iterative normalization process was stopped when the eps value, which reflected how similar the matrices in two consecutive correction steps were, dropped below 1 × 10^−4^. In addition, the filtered Hi-C reads were used to create *hic* files with the juicer tool for interactive Hi-C map inspection. The annotation of A/B Compartment of chromosome arms is available in Supplementary Data 8.

We used Hi-C maps normalized with 2 kb bins for the calculation of insulation scores, and included intrachromosomal contacts up to 50 kb in the computation^46^. Because of chromosome translocation in the *pds5a/b/c/e* plants, genomic regions at the left arm of chromosome 1 and the left arm of chromosome 5 were not included. Subsequently, potential “insulated regions” were identified by looking for regions with local insulation score minima. This is done by implementing the ‘peak’ function from the splus2R package in R with ‘span*=* 10’. In this study, we annotated regions with insulation scores lower than −0.25 and overlapping with insulation score local minima as “insulated regions”. A list containing insulation scores and insulated region annotation is available in Supplementary Data 15.

TAD-like domains that emerged on the *pds5a/b/c/e* Hi-C map were identified with the “arrowhead” algorithm, which we have previously applied to similar chromatin features in the rice and Marchantia genomes^44,47,48^. In this study, we set the cutoff for the TAD score matrix as 0.95, the minimum number of filtered pixels belonging to a potential TAD as 6, and the minimum TAD score of pixels at TAD borders as 1.05. TAD annotation is available in Supplementary Data 16. The TAD calling pipeline was not applied to pericentromeric regions because they did not show clear changes on the Hi-C map (Supplementary Fig. 1). Furthermore, Because of the translocation between chromosome 1 left arm and chromosome 5 left arm, these two chromosome arms were not subject to TAD calling (Supplementary Fig. 2).

Chromatin contacts with statistical significance were identified with the FiTHiC2 tool^31^. The resolution was set to be 2 kb, and the distance range in which the chromatin interaction model was built and chromatin loops were called was between 4 kb and 100 kb. After running FitHiC2, chromatin contacts with *q*-values less than 0.05 were used for downstream pattern analyzes.

### Chromatin immunoprecipitation and ChIP-seq data analyzes

Fourteen-day-old *Arabidopsis* leaves were fixed under vacuum for 30 minutes with 1% formaldehyde solution in MC buffer (10 mM potassium phosphate, pH 7.0, 50 mM NaCl, and 0.1 M sucrose) at room temperature. The fixation process was stopped by replacing the solution with 0.15 M glycine in MC buffer under vacuum for 10 minutes at room temperature. Approximately 0.5 gram of the fixed tissue was homogenized and suspended in nuclei isolation buffer (containing 20 mM HEPES, pH 8.0, 250 mM sucrose, 1 mM MgCl2, 5 mM KCl, 40% glycerol, 0.25% Triton X-100, 0.1 mM PMSF, and 0.1% 2-mercaptoethanol) and then filtered using double-layered miracloth (Millipore). The isolated nuclei were resuspended in 0.5 mL of sonication buffer (10 mM potassium phosphate, pH 7.0, 0.1 mM NaCl, 0.5% sarkosyl, and 10 mM EDTA). Chromatin was fragmented by sonication using a QSONICA sonicator Q800R3 to achieve an average fragment size of approximately 400 base pairs.

Next, 50 µL of 10% Triton X-100 was combined with the sonicated sample, and 25 µL of this mixture was retained as an input sample. The remaining sheared chromatin was mixed with an equal volume of IP buffer (comprising 50 mM HEPES, pH 7.5, 150 mM NaCl, 5 mM MgCl2, 10 µM ZnSO4, 1% Triton X-100, and 0.05% SDS) and incubated with antibodies (anti-Pol2, Abcam ab5408; anti-H3, Sigma H9289; anti-H3K4me3, Abcam ab8580; anti-H3K9me2, Diagenode C15410060; anti-H3K27me3, Millipore 07-449) at 4 °C for 2 h, followed by further incubation with protein A/G magnetic beads (Thermo Fisher) 4 °C for 2 h. The beads were subsequently washed at 4 °C as follows: 2 washes with IP buffer, 1 wash with IP buffer containing 500 mM NaCl, and 1 wash with LiCl buffer (0.25 M LiCl, 1% NP-40, 1% deoxycholate, 1 mM EDTA, and 10 mM Tris-HCl pH 8.0), each for 3 minutes. After a brief rinse with TE buffer (comprising 10 mM Tris-HCl, pH 8.0, and 1 mM EDTA), the beads were resuspended in 200 µL of elution buffer (containing 50 mM Tris-HCl, pH 8.0, 200 mM NaCl, 1% SDS, and 10 mM EDTA) and incubated at 65 °C for 6 h. This was followed by Proteinase K treatment at 45 °C for 1 h. DNA was purified using the MinElute PCR purification kit (QIAGEN) and subsequently converted into sequencing libraries using the NEBNext® Ultra™ II DNA Library Prep Kit (NEB).

After sequencing, ChIP-seq reads were aligned to the TAIR10 genome using Bowtie 2 (version 2.2.4). The mapped reads were used to generate coverage files with 100 bp windows using bedtools (v2.26.0). The mapped reads were analyzed by MACS2 v2.2.7.1. The “--broad” flag was on during peak calling for H3K27me3. The reads from the anti-H3 sample used as control and the other parameters are -q 0.05, -g 1.35e8. Details of enriched regions are available in Supplementary Data 9–14.

### ATAC-seq and data analyzes

ATAC-seq was performed with two biological replicates. Nuclei were extracted from 1% formaldehyde-fixed seedlings, stained with 0.5 μM DAPI, and sorted with BD Influx™ cell sorter (BD Biosciences). For each replicate, 50,000 of sorted nuclei were collected in Galbraith buffer and centrifuged at 2000 g at 4 °C for 5 min. The nuclei were resuspended with a 20 μL Tn5 transposase (Illumina) reaction. The transposed DNA was purified with MinElute PCR Purification Kit (Qiagen) and amplified with selected Nextera index oligos (Illumina). Size selection of PCR products was performed with AMPure® XP beads (Beckman Colter) to collect library molecules between 200 and 700 bp. Finally, the purified libraries were pooled and sequenced. Raw reads were mapped to the TAIR10 *Arabidopsis* reference genome with Bowtie2 and sorted with SAMtools. The mapped reads were used to generate coverage files with 100 bp windows using bedtools (v2.26.0).

### Gene expression analyzes

RNA sequencing (RNA-Seq) was performed with two biological replicates per sample. Total RNA was extracted from aerial parts of seedlings using a RNeasy Plant Mini Kit (Qiagen). Libraries were constructed with the NEBNext® Ultra™ RNA Library Prep Kit for Illumina (NEB E7770) according to manufacturer’s instructions. The RNA-Seq libraries were sequenced at Novogene (Cambridge, UK) via a Novaseq instrument in 150-bp paired-end mode. Reads were mapped to TAIR10 genome with HISAT 2 (v2.2.1) in paired-end mode. Differentially expressed genes (DEGs) were identified via the Subread and DESeq2 R packages with a cutoff of log2 fold change larger than 1.6 and false discovery rate (FDR) less than 0.01. Details of the reads count table and differentially expressed genes are available in Supplementary Data 2–7.

### Reporting summary

Further information on research design is available in the Nature Portfolio Reporting Summary linked to this article.

## Supplementary information

Supplementary Information

Peer Review file

Description of Additional Supplementary Files

Supplementary Data 1

Supplementary Data 2

Supplementary Data 3

Supplementary Data 4

Supplementary Data 5

Supplementary Data 6

Supplementary Data 7

Supplementary Data 8

Supplementary Data 9

Supplementary Data 10

Supplementary Data 11

Supplementary Data 12

Supplementary Data 13

Supplementary Data 14

Supplementary Data 15

Supplementary Data 16

Reporting Summary

## Source data

Source Data

## Data Availability

Data supporting the findings of this work are available within the paper and its Supplementary Information files. A reporting summary for this article is available as a Supplementary Information file. Short read data of in situ Hi-C, ChIP-seq, ATAC-seq, and RNA-seq are publicly available at NCBI Sequence Read Archive under accession number PRJNA1043456. Large datasets, such as normalized Hi-C matrices and BigWig ChIP-seq and ATAC-seq track files are available in the Figshare repository [10.6084/m9.figshare.24533263.v1]. The epigenetic profile of various histone marks is available from our previous publication^43^. Source data are provided with this paper.

## Code availability

The code for Hi-C read mapping, filtering, and matrix normalization is available in the GitHub repository [https://github.com/changliu325/Arabidopsis_crwn1_chromatin/tree/master/HiC]. The code for TAD-like domain calling and insulation score computation is also available in the GitHub repository [https://github.com/changliu325/pds5_chromatin].
