# Supplementary material for: Mutations of PDS5 genes enhance TAD-like domain formation in Arabidopsis thaliana
Source: Nat Commun. 2024 Oct 29;15:9308. doi: 10.1038/s41467-024-53760-x (PMC11519323; doi:10.1038/s41467-024-53760-x)
Supplement: Supplementary file 1 — Supplementary Information [file 41467_2024_53760_MOESM1_ESM.pdf]

Mutations of *PDS5* genes enhance TAD-like domain formation in *Arabidopsis thaliana*

Göbel *et al.*

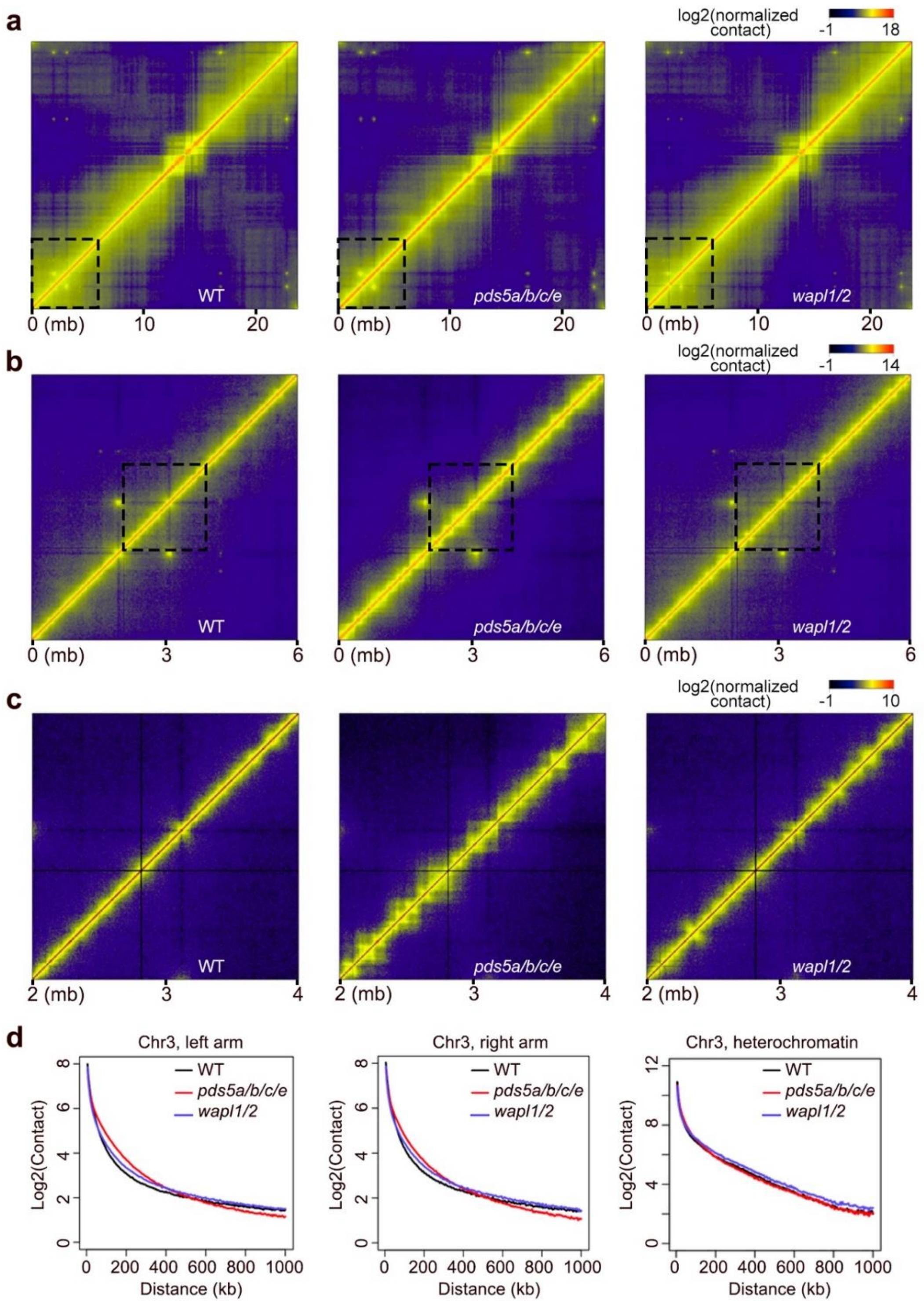

Supplementary Figure 1. Comparison of Hi-C maps of wild-type, *pds5*, and *wapl* mutants. (a) Hi-C maps from chromosome 3 normalized at 100 kb resolution. The color key represents normalized contact strength. The black square shows the respective zoomed-in section (b, c) that can be seen in the following panels showing Hi-C maps normalized at 20 and 5 kb, respectively. (d) Decay of chromatin contact frequency along with increasing genomic distance. The centromeric and pericentromeric regions are collectively referred to as heterochromatin.

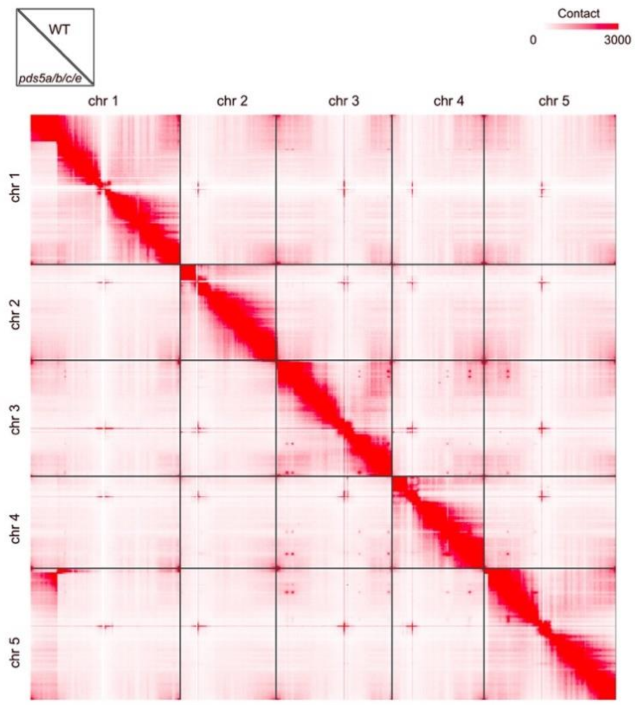

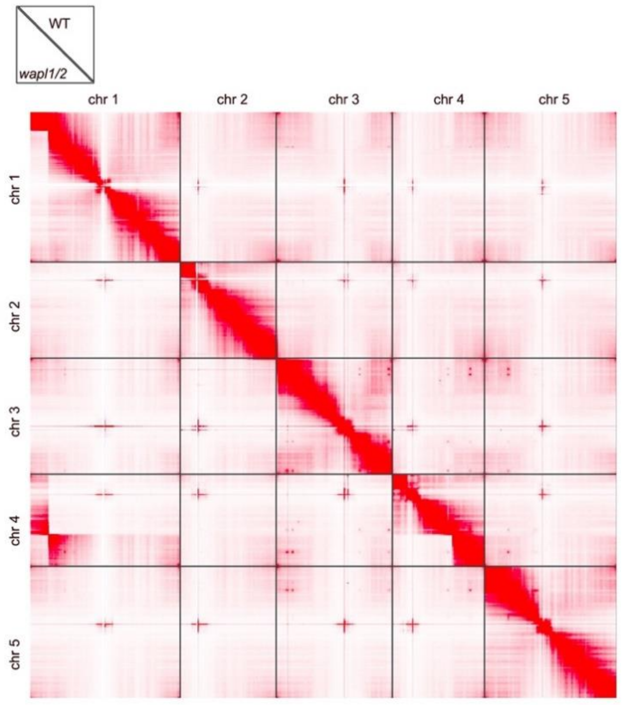

Supplementary Figure 2. Comparison of Hi-C maps between the *pds5a/b/c/e* and *wapl1/2* mutant and the wild-type. The mutant *pds5a/b/c/e* shows inter-chromosomal rearrangement between chromosome 1 and chromosome 5, and *wapl1/2* between chromosome 1 and chromosome 4.

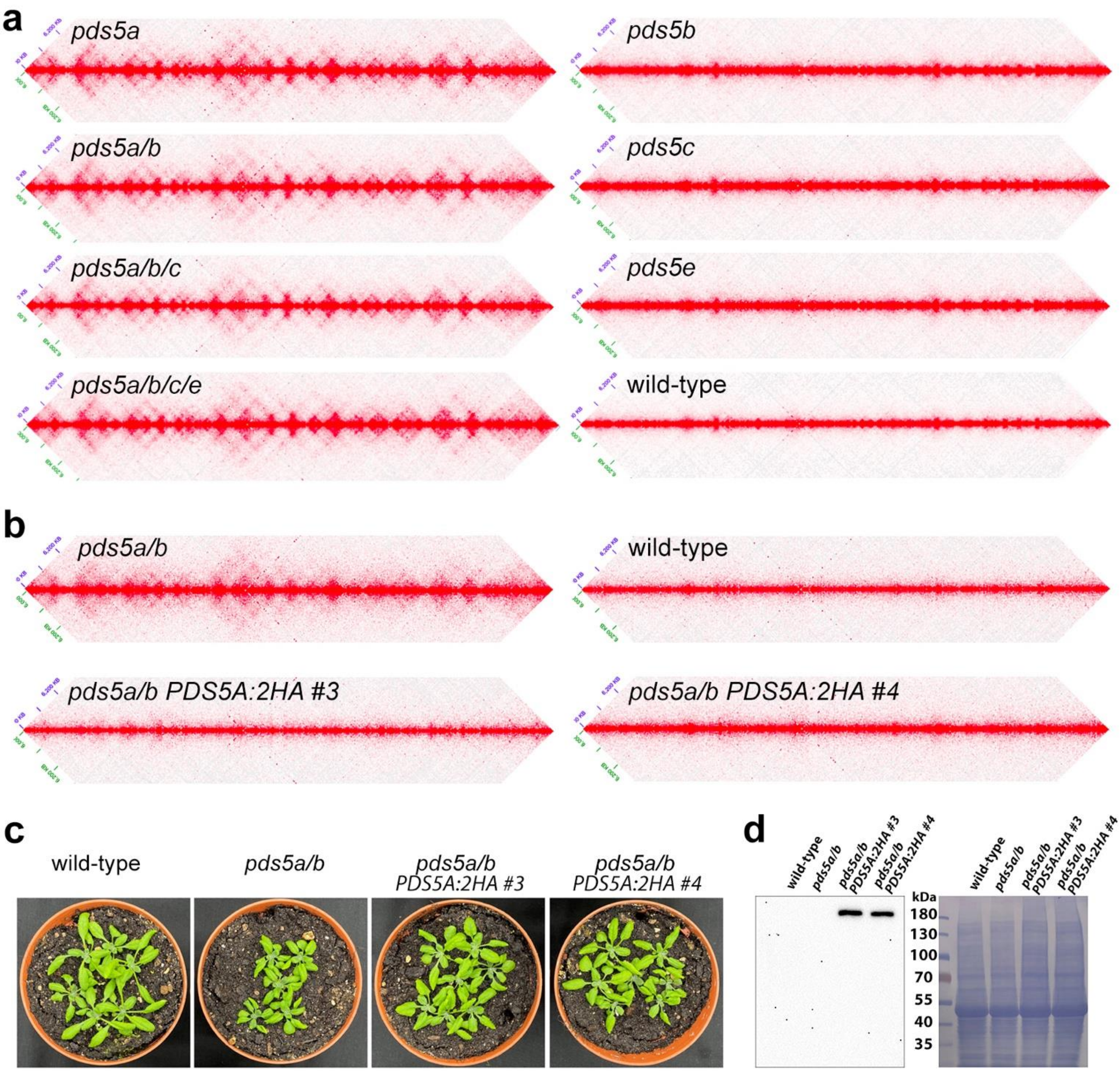

Supplementary Figure 3. PDS5A is the main PDS5 homolog that suppresses TAD-like domains. (a) Hi-C maps of various single and higher-order *pds5* mutants reveal the emergence of TADs upon losing *PDS5A*. Individual Hi-C maps show the same 1.5 Mb region from chromosome 5. (b,c) Complementation of *pds5a/b* chromatin organization phenotype (b) and seedling growth phenotype (c) by *PDS5A*. In panel (b), Hi-C maps of the 1.5 Mb genomic region, as shown (a), are illustrated. (d) Detection of PDS5A:2HA protein expression. The membrane was probed with anti-HA antibody (left) and stained afterward with Coomassie Blue (right). N=1. Source data are provided as a Source Data file.

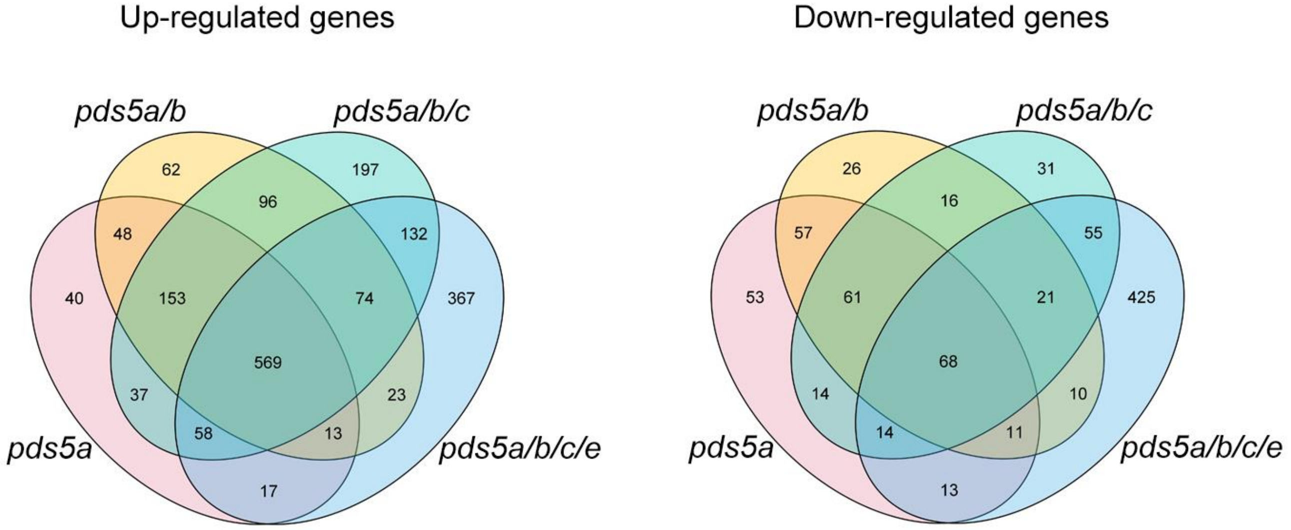

Supplementary Figure 4. Venn diagrams of differentially expressed genes in various *pds5* mutants.

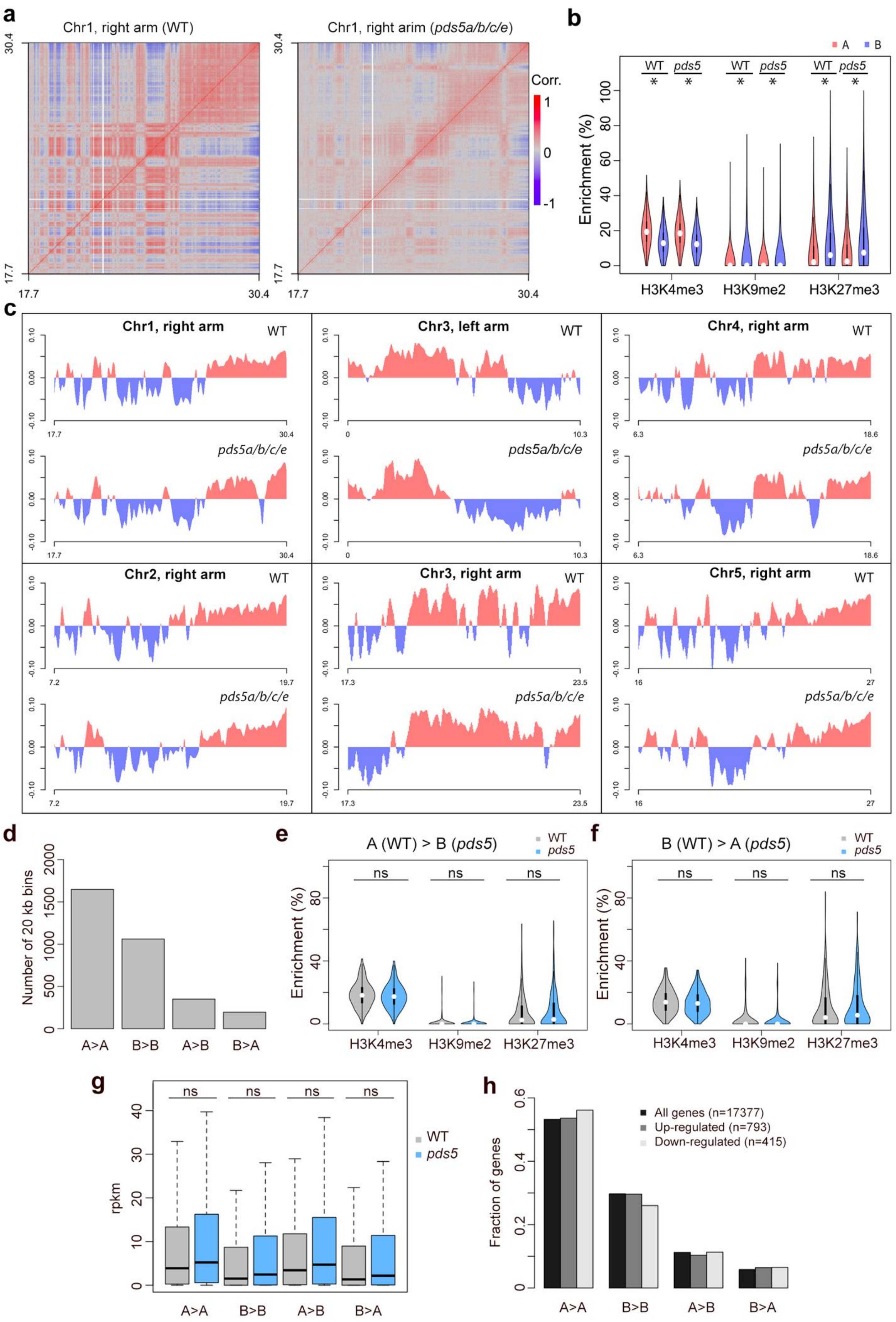

a
Chr1, right arm (WT)
Chr1, right arim (pds5a/b/c/e)
Corr.
b
H3K4me3
H3K9me2
H3K27me3
Enrichment (%)
c
Chr1, right arm
Chr3, left arm
Chr4, right arm
Chr2, right arm
Chr3, right arm
Chr5, right arm
WT
pds5a/b/c/e
d
Number of 20 kb bins
A>A
B>B
A>B
B>A
e
A (WT) > B (pds5)
f
B (WT) > A (pds5)
ns
g
rpkm
WT
pds5
h
Fraction of genes
All genes (n=17377)
Up-regulated (n=793)
Down-regulated (n=415)

Supplementary Figure 5. Comparison of non- and switching A/B compartment intervals and gene expression in *pds5a/b/c/e* mutant. (a) Correlation matrix of Hi-C map from chromosome 1 right arm of *pds5a/b/c/e* and wild-type plants. (b) Differential deposition of histone marks in A/B compartments in each genotype. *pds5* refers to *pds5a/b/c/e*. *, significant ($p < 10^{-16}$) according to a two-sided Mann–Whitney U-tests. (c) A/B compartment annotation in wild-type and *pds5a/b/c/e*. (d) Sizes of non- and switching A/B compartment intervals in *pds5a/b/c/e*. The annotation "X>Y" below each column indicates compartment annotation in wild-type ("X") and *pds5a/b/c/e* ("Y"). (e,f) Comparison of histone marks in regions switching A/B compartment identities in wild-type and *pds5a/b/c/e* plants. *pds5* refers to *pds5a/b/c/e*. ns, not significant according to two-sided Mann–Whitney U-tests. (g) Expression of genes located in the A/B sharing and switching compartment of each genotype. *pds5* refers to *pds5a/b/c/e*. ns, not significant according to two-sided Mann–Whitney U-tests. (h) Distribution of DEGs in the non- and switching A/B compartments in *pds5a/b/c/e*.

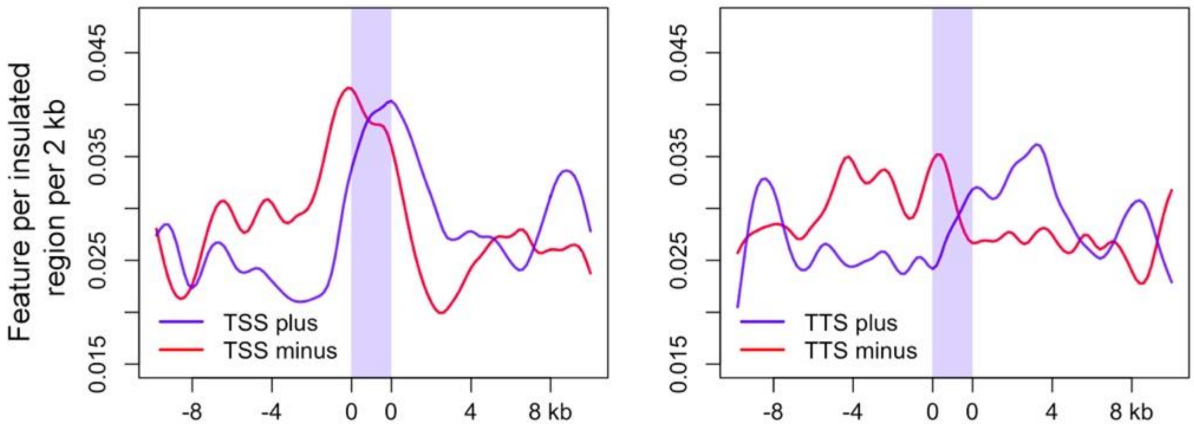

Supplementary Figure 6. Distribution of TSS and TTS at insulated chromatin regions (violet bar) identified in *pds5a/b/c/e*. The left panel shows the distribution of TSS, and the right panel shows the distribution of TTS.

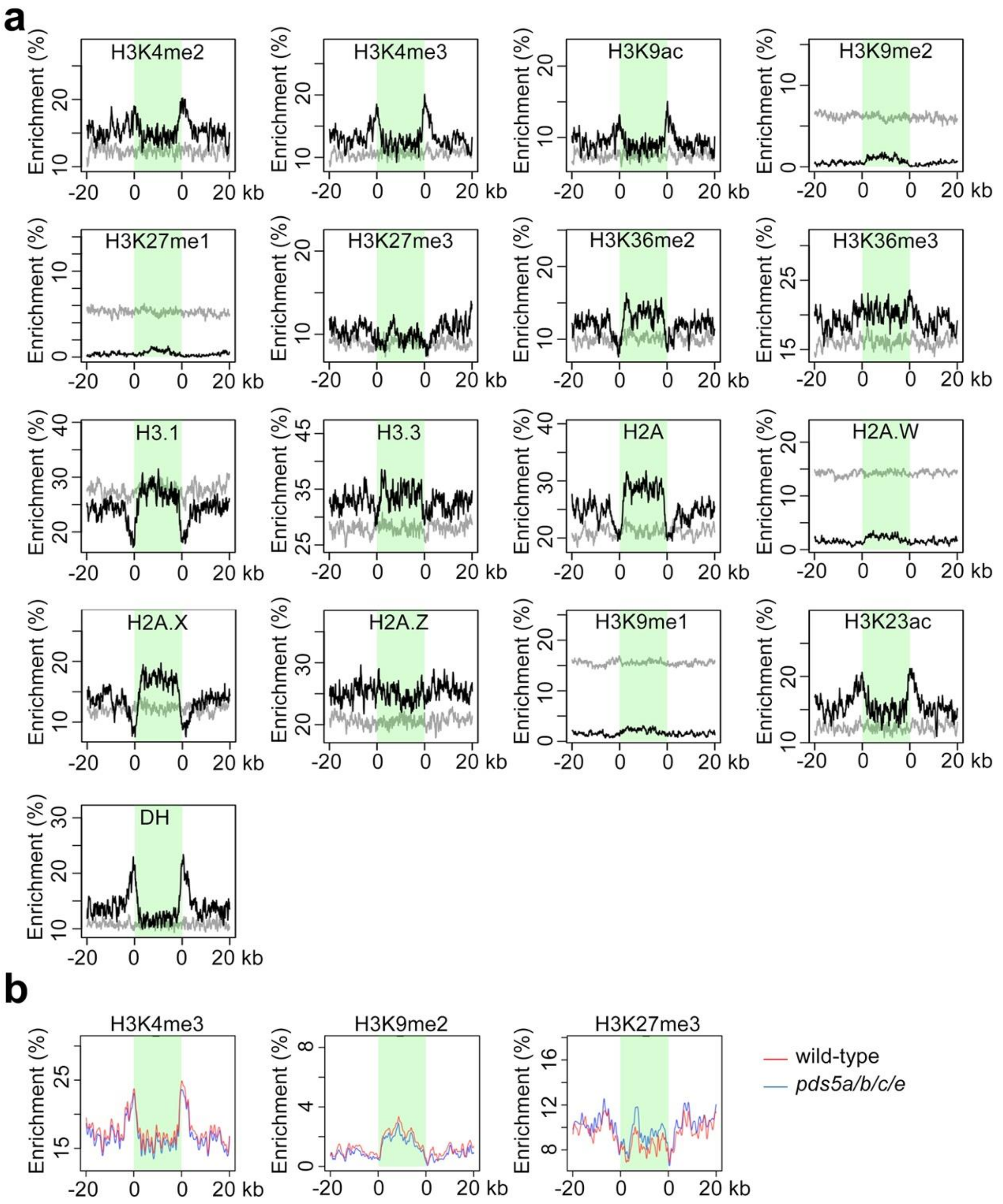

Supplementary Figure 7. Epigenetic marks and chromatin accessibility across TAD-like domains. (a) For each plot, part of the curve describing TAD-like domains was linearly transformed to align the domain borders. The grey curve represents the background, which was estimated by redistributing TAD-like domains randomly throughout the genome. The black curve depicts an epigenetic histone modification, a histone variate, or chromatin accessibility (DH). These profiles are accessible through our previous work[1]. (b) Comparison of euchromatin (H3K4me3), heterochromatin (H3K9me2), and facultative heterochromatin (H3K27me3) epigenetic marks in wild-type and *pds5a/b/c/e*. These profiles were generated in this study by using the corresponding plant materials.

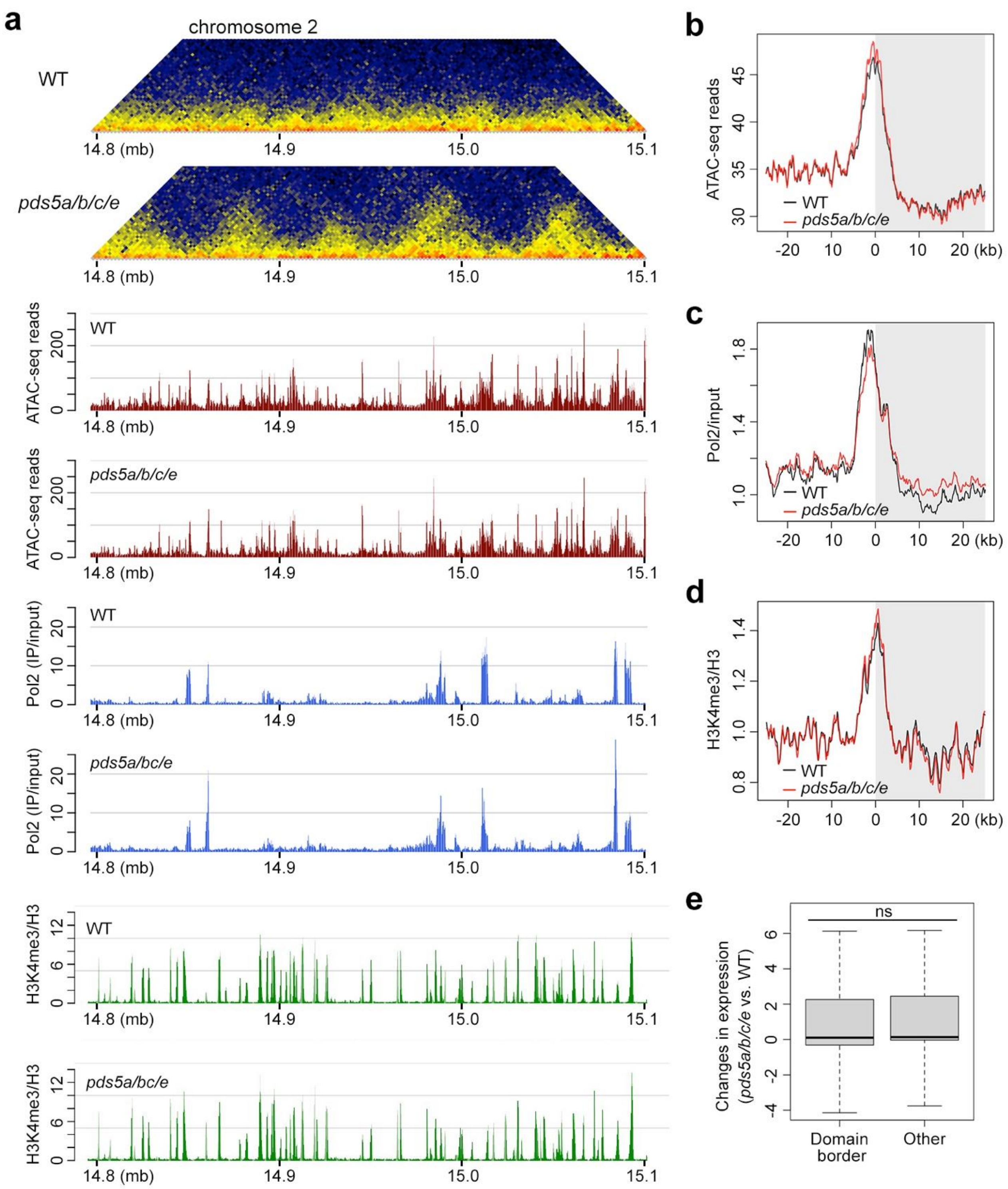

Supplementary Figure 8. Correlation between TADs, chromatin accessibility and gene expression between wild-type and *pds5a/b/c/e* mutant. (a) Comparison of Hi-C maps from a 0.3 Mb genomic region at chromosome 2 with ATAC-seq and Pol II ChIP-seq data. (b-d) Comparison of ATAC-seq reads (b), Pol2 (c), and H3K4me3 (d) in wild-type and *pds5a/b/c/e* around TAD-like domain borders that emerged in *pds5a/b/c/e*. (e) Changes in the gene expression of genes located at TAD-like domain boundaries and other genes across the whole genome. ns, not significant according to a two-sided Mann–Whitney U-test.

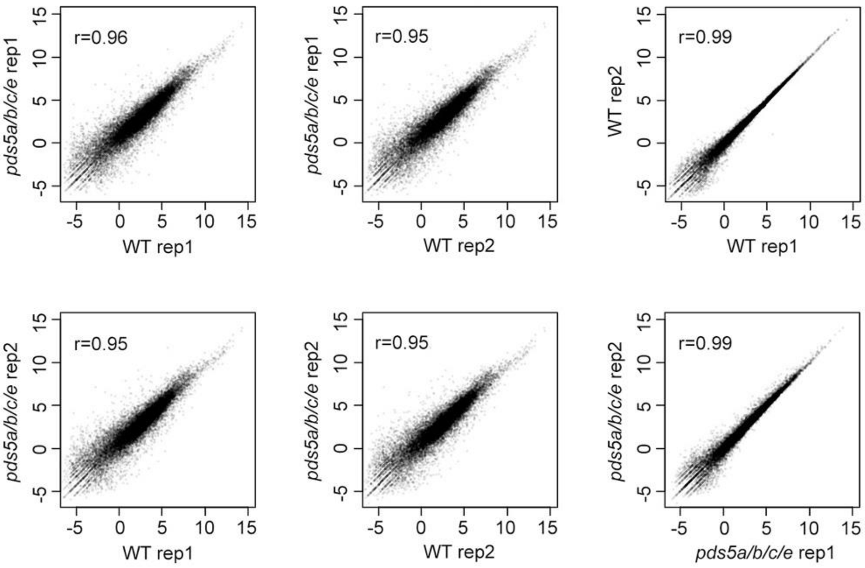

Supplementary Figure 9. Comparison of gene expression (log2(rpkm)) among individual wild-type and *pds5a/b/c/e* samples. r values depict Pearson correlation coefficients.

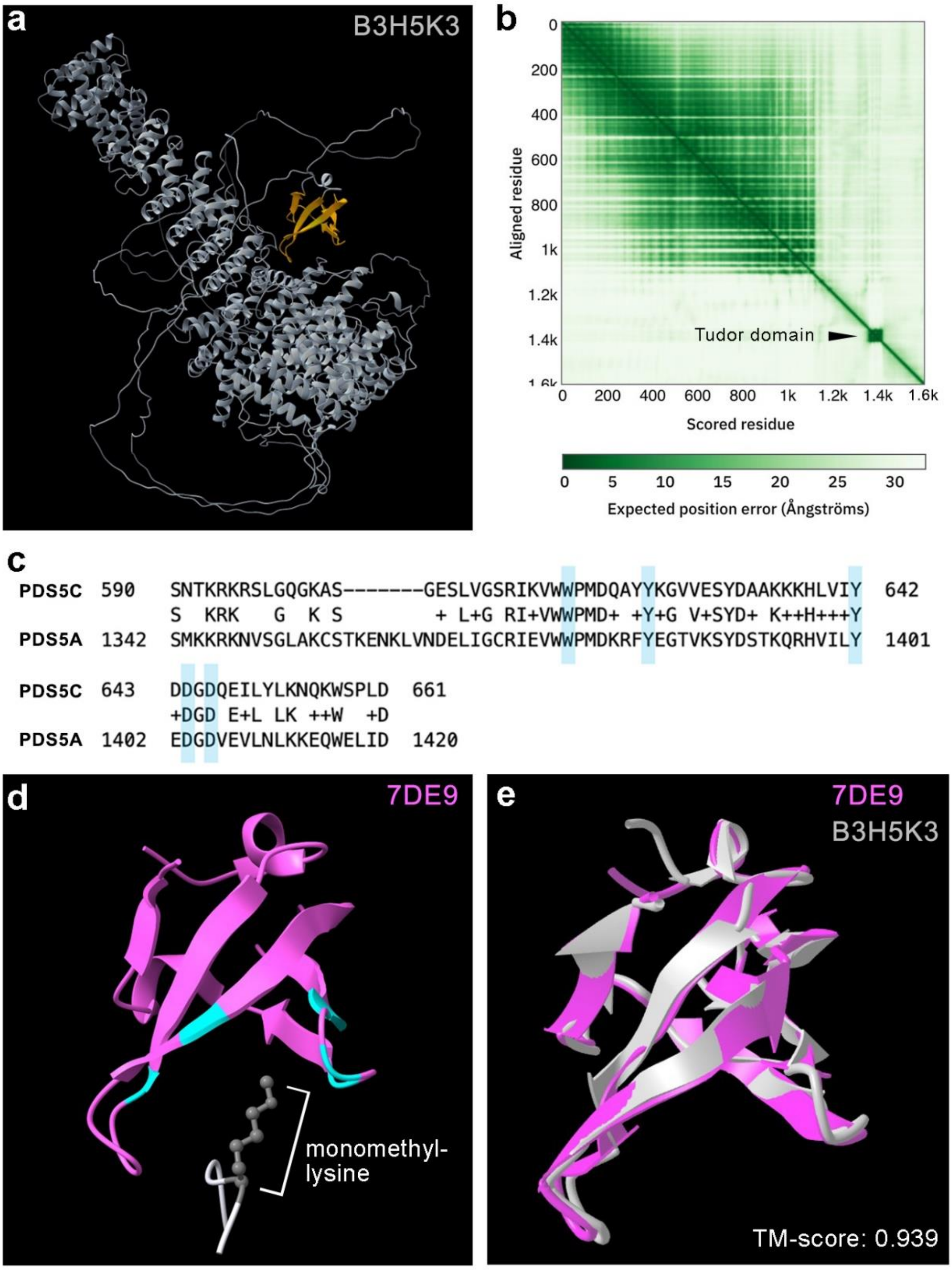

Supplementary Figure 10. Analysis of the PDS5A Tudor domain structure. (a,b) Alphafold prediction of the PDS5A protein (UniProt identifier: B3H5K3). The yellow structure in (a) depicts the Tudor domain. (c) Sequence alignment of Tudor domain regions in PDS5A and PDS5C. The highlighted residues are involved in interactions with methylated H3K4me1 peptide according to the crystal structure[2] (PDB identifier: 7DE9). (d) Crystal structure showing the interaction between PDS5C Tudor domain and H3K4me1 peptide[2]. (e) Alphafold-predicted PDS5A Tudor domain superimposed onto the experimentally determined counterpart in PDS5C.

Supplementary references

1 Hu, B. *et al.* Plant lamin-like proteins mediate chromatin tethering at the nuclear periphery. *Genome Biol* **20**, 87 (2019).

2 Niu, Q. *et al.* A histone H3K4me1-specific binding protein is required for siRNA accumulation and DNA methylation at a subset of loci targeted by RNA-directed DNA methylation. *Nat Commun* **12**, 3367 (2021).
