# Supplementary material for: Mutations of PDS5 genes enhance TAD-like domain formation in Arabidopsis thaliana
Source: Nat Commun. 2024 Oct 29;15:9308. doi: 10.1038/s41467-024-53760-x (PMC11519323; doi:10.1038/s41467-024-53760-x)
Supplement: Supplementary file 3 — Description of Additional Supplementary Files [file 41467_2024_53760_MOESM3_ESM.pdf]

Description of Additional Supplementary Files

File Name: Supplementary Data 1
Description: Information of the sequenced HiC libraries.

File Name: Supplementary Data 2
Description: The count table of the RNA-seq analysis in indicated genotypes.

File Name: Supplementary Data 3
Description: The RPKM value of genes in the RNA-seq analysis in indicated genotypes.

File Name: Supplementary Data 4
Description: Identified differentially-expressed genes in *pds5a* compared to WT.

File Name: Supplementary Data 5
Description: Identified differentially-expressed genes in *pds5ab* compared to WT.

File Name: Supplementary Data 6
Description: Identified differentially-expressed genes in *pds5abc* compared to WT.

File Name: Supplementary Data 7
Description: Identified differentially-expressed genes in *pds5abce* compared to WT.

File Name: Supplementary Data 8
Description: Identification of AB compartments in WT and *pds5a/b/c/e.*

File Name: Supplementary Data 9
Description: Identified enriched regions with H3K4me3 in WT.

File Name: Supplementary Data 10
Description: Identified enriched regions with H3k9me2 in WT.

File Name: Supplementary Data 11
Description: Identified enriched regions with H3K27me3 in WT.

File Name: Supplementary Data 12
Description: Identified enriched regions with H3K4me3 in *pds5a/b/c/e*.

File Name: Supplementary Data 13
Description: Identified enriched regions with H3k9me2 in *pds5a/b/c/e.*

File Name: Supplementary Data 14
Description: Identified enriched regions with H3K27me3 in *pds5a/b/c/e.*

File Name: Supplementary Data 15
Description: Insulation scores in WT and *pds5a/b/c/e.*

File Name: Supplementary Data 16
Description: Identified TAD-like domains in *pds5a/b/c/e.*
