# Supplementary material for: Mutations of PDS5 genes enhance TAD-like domain formation in Arabidopsis thaliana
Source: Nat Commun. 2024 Oct 29;15:9308. doi: 10.1038/s41467-024-53760-x (PMC11519323; doi:10.1038/s41467-024-53760-x)
Supplement: Supplementary file 20 — Reporting Summary [file 41467_2024_53760_MOESM20_ESM.pdf]

nature portfolio

Corresponding author(s): *Double-blind peer review submissions: write DBPR and your manuscript number here instead of author names.*

Last updated by author(s): Oct 14, 2024

Reporting Summary

Nature Portfolio wishes to improve the reproducibility of the work that we publish. This form provides structure for consistency and transparency in reporting. For further information on Nature Portfolio policies, see our Editorial Policies and the Editorial Policy Checklist.

Statistics

For all statistical analyses, confirm that the following items are present in the figure legend, table legend, main text, or Methods section.

n/a	Confirmed	
☐	☒	The exact sample size (*n*) for each experimental group/condition, given as a discrete number and unit of measurement
☐	☒	A statement on whether measurements were taken from distinct samples or whether the same sample was measured repeatedly
☐	☒	The statistical test(s) used AND whether they are one- or two-sided<br>*Only common tests should be described solely by name; describe more complex techniques in the Methods section.*
☒	☐	A description of all covariates tested
☒	☐	A description of any assumptions or corrections, such as tests of normality and adjustment for multiple comparisons
☐	☒	A full description of the statistical parameters including central tendency (e.g. means) or other basic estimates (e.g. regression coefficient) AND variation (e.g. standard deviation) or associated estimates of uncertainty (e.g. confidence intervals)
☐	☒	For null hypothesis testing, the test statistic (e.g. *F*, *t*, *r*) with confidence intervals, effect sizes, degrees of freedom and *P* value noted<br>*Give P values as exact values whenever suitable.*
☒	☐	For Bayesian analysis, information on the choice of priors and Markov chain Monte Carlo settings
☒	☐	For hierarchical and complex designs, identification of the appropriate level for tests and full reporting of outcomes
☐	☒	Estimates of effect sizes (e.g. Cohen's *d*, Pearson's *r*), indicating how they were calculated

Our web collection on statistics for biologists contains articles on many of the points above.

Software and code

Policy information about availability of computer code

Data collection	All sequencing samples generated in this study were prepared in house and sequenced on Illumina platforms.
Data analysis	Bowtie 2 (v2.2.4)<br>HISAT 2<br>R (v4.1.0)<br>macs2<br>Detailed parameters are described in relative sections in "Methods".

For manuscripts utilizing custom algorithms or software that are central to the research but not yet described in published literature, software must be made available to editors and reviewers. We strongly encourage code deposition in a community repository (e.g. GitHub). See the Nature Portfolio guidelines for submitting code & software for further information.

Data

Policy information about availability of data

All manuscripts must include a data availability statement. This statement should provide the following information, where applicable:

- Accession codes, unique identifiers, or web links for publicly available datasets
- A description of any restrictions on data availability
- For clinical datasets or third party data, please ensure that the statement adheres to our policy

Short read data of in situ Hi-C, ChIP-seq, ATAC-seq, and RNA-seq are publicly available at NCBI Sequence Read Archive under accession number PRJNA1043456. Large datasets, such as Hi-C matrices, ChIP-seq and ATAC-seq track files (BigWig format) are available in the figshare repository, which are accessible with the following link: https://figshare.com/s/8a653d26c484048b67bf with a reserved Digital Object Identifier (DOI) 10.6084/m9.figshare.24533263.

Human research participants

Policy information about studies involving human research participants and Sex and Gender in Research.

Reporting on sex and gender	not applicable
Population characteristics	not applicable
Recruitment	not applicable
Ethics oversight	not applicable

Note that full information on the approval of the study protocol must also be provided in the manuscript.

Field-specific reporting

Please select the one below that is the best fit for your research. If you are not sure, read the appropriate sections before making your selection.

☒ Life sciences ☐ Behavioural & social sciences ☐ Ecological, evolutionary & environmental sciences

For a reference copy of the document with all sections, see nature.com/documents/nr-reporting-summary-flat.pdf

Life sciences study design

All studies must disclose on these points even when the disclosure is negative.

Sample size	No sample-size calculation was performed for each replicate. For ChIP, gene expression, ATAC-seq, RNA-seq and Hi-C experiments, the weight of each batch of harvested samples was approximately 0.5g, consisting of at least 50 seedlings, which was required as the starting material. For these experiments, such a sample size is widely accepted by the Arabidopsis research community.
Data exclusions	No data was excluded from the analyses, except for genomic regions with poor mappability in Hi-C analysis.
Replication	ATAC-seq data: 2 biological replictes; ChIP-seq data: 2 biological replicates; RNA-seq data: 2 biological replicates; Hi-C: 2 biological replicates, except for control Hi-C data shown in FigS3 which has one biological replicate. All the attempts at replication were successful. Plants in different replications were grown in batches at least separated by two weeks.
Randomization	Plants with identical sample identity were grown in at least three pots or on three half-strength MS medium plates, which were placed randomly in the growth chamber.<br>For sample harvesting, randomization was also applied, in which control- and heat-stressed plants of the same genotype were randomly chosen from the corresponding pots or medium plates.
Blinding	Blinding was not applicable. For both the experiments in wet lab and NGS data analyses, we applied identical protocol and pipeline to individual samples, respectively.

Reporting for specific materials, systems and methods

We require information from authors about some types of materials, experimental systems and methods used in many studies. Here, indicate whether each material, system or method listed is relevant to your study. If you are not sure if a list item applies to your research, read the appropriate section before selecting a response.

Materials & experimental systems

n/a	Involved in the study
☐	☒ Antibodies
☒	☐ Eukaryotic cell lines
☒	☐ Palaeontology and archaeology
☒	☐ Animals and other organisms
☒	☐ Clinical data
☒	☐ Dual use research of concern

Methods

n/a	Involved in the study
☐	☒ ChIP-seq
☒	☐ Flow cytometry
☒	☐ MRI-based neuroimaging

Antibodies

Antibodies used

anti-Pol2: Abcam ab5408
anti-H3 (N-terminal): Sigma H9289, dilution:1/
anti-H3K4me3: Abcam ab8580
anti-H3K9me2: Diagenode C15410060
antiH3K27me3: Millipore 07-449

Validation

The antibodies have been validated by the vendor:

https://www.abcam.com/products/primary-antibodies/rna-polymerase-ii-ctd-repeat-ysptsps-phospho-s5-antibody-4h8-chip-grade-ab5408.html

ChIP-seq

Data deposition

☒ Confirm that both raw and final processed data have been deposited in a public database such as GEO.

☐ Confirm that you have deposited or provided access to graph files (e.g. BED files) for the called peaks.

Data access links
May remain private before publication.

Short read data of ChIP-seq is publicly available at NCBI Sequence Read Archive under accession number PRJNA1043456.

Processed ChIP-seq files describing reads distribution across the genome can be found at:

https://figshare.com/s/8a653d26c484048b67bf

Files in database submission

In the above-mentioned figshare link, the BigWig files of each datasets are available for downloading:
At_col_IP_rep1_SeqDepthNorm.bw: ChIP-seq, coverage file Wild-type, anti-Pol2, replicate 1
At_col_Input_rep1_SeqDepthNorm.bw: ChIP-seq, coverage file Wild-type, input, replicate 1
At_pds5_IP_rep1_SeqDepthNorm.bw: ChIP-seq, coverage file pds5a/b/c/e, anti-Pol2, replicate 1
At_pds5_Input_rep1_SeqDepthNorm.bw: ChIP-seq, coverage file pds5a/b/c/e, input, replicate 1
At_WT_Pol2_IP_rep2_SeqDepthNorm.bw: ChIP-seq, coverage file Wild-type, anti-Pol2, replicate 2
At_WT_Pol2_input_rep2_SeqDepthNorm.bw: ChIP-seq, coverage file Wild-type, input, replicate 2
At_pds5_Pol2_IP_rep2_SeqDepthNorm.bw: ChIP-seq, coverage file pds5a/b/c/e, anti-Pol2, replicate 2
At_pds5_Pol2_input_rep2_SeqDepthNorm.bw: ChIP-seq, coverage file pds5a/b/c/e, input, replicate 2

C_H3_2.bw: ChIP-seq, coverage file Wild-type, anti-H3, replicate 1
C_K4_2.bw: ChIP-seq, coverage file Wild-type, anti-H3K4me3, replicate 1
C_K27_1.bw: ChIP-seq, coverage file Wild-type, anti-H3K27me3, replicate 1
P_H3_1.bw: ChIP-seq, coverage file pds5a/b/c/e, anti-H3, replicate 1
P_K4_1.bw: ChIP-seq, coverage file pds5a/b/c/e, anti-H3K4me3, replicate 1
P_K27_2.bw: ChIP-seq, coverage file pds5a/b/c/e, anti-H3K27me3, replicate 1
C_H3_3.bw: ChIP-seq, coverage file Wild-type, anti-H3, replicate 2
C_K4_3.bw: ChIP-seq, coverage file Wild-type, anti-H3K4me3, replicate 2
C_K27_2.bw: ChIP-seq, coverage file Wild-type, anti-H3K27me3, replicate 2
P_H3_3.bw: ChIP-seq, coverage file pds5a/b/c/e, anti-H3, replicate 2
P_K4_3.bw: ChIP-seq, coverage file pds5a/b/c/e, anti-H3K4me3, replicate 2
P_K27_3.bw: ChIP-seq, coverage file pds5a/b/c/e, anti-H3K27me3, replicate 2

Col_H3.bw: ChIP-seq, coverage file Wild-type, anti-H3, replicate 1
Col_K9.bw: ChIP-seq, coverage file Wild-type, anti-H3K9me2, replicate 1
pds5_1_H3.bw: ChIP-seq, coverage file pds5a/b/c/e, anti-H3, replicate 1
pds5_1_K9.bw: ChIP-seq, coverage file pds5a/b/c/e, anti-H3K9me2, replicate 1
Col_2_H3.bw: ChIP-seq, coverage file Wild-type, anti-H3, replicate 2
Col_2_H3K9.bw: ChIP-seq, coverage file Wild-type, anti-H3K9me2, replicate 2
pds5_2_H3.bw: ChIP-seq, coverage file pds5a/b/c/e, anti-H3, replicate 2
pds5_2_K9.bw: ChIP-seq, coverage file pds5a/b/c/e, anti-H3K9me2, replicate 2

Genome browser session (e.g. UCSC)	not available

Methodology

Replicates	2 biological replicates
Sequencing depth	All samples were sequenced in PE150 mode, and they had at least 10,000,000 reads after read mapping.
Antibodies	anti-Pol2: Abcam ab5408, dilution: 2ul/300ul (https://www.abcam.com/en-us/products/primary-antibodies/rna-polymerase-ii-ctd-repeat-ysptsps-phospho-s5-antibody-4h8-chip-grade-ab5408)<br>anti-H3 (N-terminal): Sigma H9289, batch:0000085839, dilution: 1ul/100ul chromatin (https://www.sigmaaldrich.com/DE/de/search/h9289?focus=products&page=1&perpage=30&sort=relevance&term=h9289&type=product)<br>anti-H3K4me3: Abcam ab8580, LOT:GR3458434-1, dilution: 3ul/300ul chromatin (https://www.abcam.com/en-us/products/primary-antibodies/histone-h3-tri-methyl-k4-antibody-chip-grade-ab8580)<br>anti-H3K9me2: Diagenode C15410060, LOT: A90-0042, dilution: 3ul/300ul chromatin (https://www.diagenode.com/en/p/h3k9me2-polyclonal-antibody-classic-50-ug-44-ul)<br>antiH3K27me3: Millipore 07-449, LOT:3517402, dilution: 3ul/300ul chromatin (https://www.merckmillipore.com/DE/de/product/Anti-trimethyl-Histone-H3-Lys27-Antibody,MM_NF-07-449)
Peak calling parameters	Peak calling was not applied for anti-Pol2.<br>For H3K4me3, H3K9me2, and H3K27me3 , the following peak calling parameters were used:<br>macs2 callpeak -t K4.bam -c H3.bam -f BAMPE -q 0.05 -g 1.35e8<br>macs2 callpeak -t K9.bam -c H3.bam -f BAMPE -q 0.05 -g 1.35e8<br>macs2 callpeak -t K27.bam -c H3.bam -f BAMPE -q 0.05 -g 1.35e8 --broad
Data quality	*Describe the methods used to ensure data quality in full detail, including how many peaks are at FDR 5% and above 5-fold enrichment.*
Software	*Describe the software used to collect and analyze the ChIP-seq data. For custom code that has been deposited into a community repository, provide accession details.*
