# Supplementary material for: Mutations of PDS5 genes enhance TAD-like domain formation in Arabidopsis thaliana
Source: Nat Commun. 2024 Oct 29;15:9308. doi: 10.1038/s41467-024-53760-x (PMC11519323; doi:10.1038/s41467-024-53760-x)
Supplement: Supplementary file 2 — Peer Review file [file 41467_2024_53760_MOESM2_ESM.pdf]

nature portfolio

Peer Review File

Mutations of PDS5 genes enhance TAD-like domain formation in Arabidopsis thaliana

Corresponding Author: Dr Hua Jiang

This manuscript has been previously reviewed at another journal. This document only contains reviewer comments, rebuttal and decision letters for versions considered at Nature Communications.

This file contains all reviewer reports in order by version, followed by all author rebuttals in order by version.

Version 0:

Reviewer comments:

Reviewer #1

(Remarks to the Author)
My comments are the same as the previous ones I gave nature plants. It is a technically sound paper. Although I think it lack novelty, but it is up to the editor to decide.

Open Access This Peer Review File is licensed under a Creative Commons Attribution 4.0 International License, which permits use, sharing, adaptation, distribution and reproduction in any medium or format, as long as you give appropriate credit to the original author(s) and the source, provide a link to the Creative Commons license, and indicate if changes were made.
In cases where reviewers are anonymous, credit should be given to 'Anonymous Referee' and the source.
The images or other third party material in this Peer Review File are included in the article's Creative Commons license,

unless indicated otherwise in a credit line to the material. If material is not included in the article's Creative Commons license and your intended use is not permitted by statutory regulation or exceeds the permitted use, you will need to obtain permission directly from the copyright holder.
To view a copy of this license, visit https://creativecommons.org/licenses/by/4.0/

Response to review comments

Reviewer #1:

Comments: My comments are the same as the previous ones I gave nature plants. It is a technically sound paper. Although I think it lacks novelty, but it is up to the editor to decide.

Answer: We appreciate your comments.
